# Comparison of fertility results after vaginal insemination using different thawing procedures and packages for frozen ram semen

**DOI:** 10.1186/1751-0147-49-26

**Published:** 2007-09-28

**Authors:** Heiko Paulenz, Tormod Ådnøy, Lennart Söderquist

**Affiliations:** 1 Team Semin, Norwegian School of Veterinary Science, PO Box 8146 Dep., N-0033 Oslo, Norway; 2 Department of Production Animal Clinical Sciences, Norwegian School of Veterinary Science, PO Box 8146 Dep., N-0033 Oslo, Norway; 3 Department of Animal and Aquacultural Sciences, Norwegian University of Life Sciences, PO Box 5003, N-1432 Ås, Norway; 4 Department of Clinical Sciences, Division of Reproduction, Faculty of Veterinary Medicine and Animal Science, Swedish University of Agricultural Sciences, PO Box 7054 Ultuna, SE-75007 Uppsala, Sweden

## Abstract

**Background:**

The effect of different thawing procedures for ram semen frozen in minitubes and mini straws on the fertility of sheep was tested in a field trial.

**Methods:**

Altogether, 719 Norwegian Crossbred ewes, aged between six months and six-and-a-half years from 8 farms, were inseminated vaginally in natural oestrus with frozen-thawed semen. Minitubes were thawed at 70°C for 8 sec (T70) and mini straws either at 50°C for 9 sec (S50) or at 35°C for 12 sec (S35).

**Results:**

Vaginal insemination with 200 × 10^6 ^spermatozoa resulted in 25-days non-return rates of 63.2, 59.6, and 62.5% (overall 61.8%), respectively, and lambing rates of 56.8, 55.0, and 59.2% (overall 57.0%), respectively. No significant effect on fertility (as 25-days non-return- or lambing rate) was seen for straw type/thawing temperature (P = 0.5/0.5), but semen filled in mini straws and thawed at 35°C resulted numerically in the highest lambing rate (59.2%). A significant effect was, however, seen for farmer (P = >0.0001/>0.0001) and ram (P = 0.009/0.002). Moreover, age of the ewes had a significant effect on the NR rate (0.007), but not on lambing rate (P = 0.2).

**Conclusion:**

A vaginal deposition of frozen ram semen containing approximately 200 × 10^6 ^spermatozoa, filled in mini straws and thawed at 35°C is a simplified technique that under field conditions and used on a do-it-yourself regime gives acceptable lambing rates in Norway.

## Background

The fertility of Norwegian Crossbred ewes is very high, with natural mating being predominant. Because the average flock size is only approximately 52 adult animals, a cooperative scheme for breeding has been developed. During the mating season rams are moved between farms that belong to the same "ram circle" or breeding group, according to their breeding requirements [1,2].

In the 1960s cervical insemination with frozen-thawed ram semen was developed in Norway [3] for routine use in artificial insemination (AI). At that time AI was introduced as a supplement to natural mating and therefore only less than one per cent of the ewes were inseminated artificially over the next three decades. The routine cryopreservation protocol developed during this period included manually filling/sealing and freezing in minitubes (0.25 ml, Minitüb GMBH, Tiefenbach, Germany), and subsequent thawing at 70°C (±2°C) for 8 sec. The recommendation that thawing of ram semen should be fast (few seconds) and consequently at a relatively high temperature (70°C) was based upon the results obtained by Aamdal and Andersen [4,5]. They found a considerably better semen quality, measured as sperm motility and sperm membrane integrity, after thawing of semen in 0.25 ml PVC straws, which were similar in shape and thickness to minitubes, at 70°C for 8 sec compared with 35°C for 15 or 30 sec using straws. It is noteworthy that semen at that time was frozen at lower concentrations (500 × 10^6^/ml) compared to the routines used in the last three decades (1000 × 10^6^/ml). This difference might have implications on semen quality after freezing and thawing.

In the middle of the 1990s the Norwegian animal health authorities issued several restrictions on the movement of sheep between flocks, in order to control the emergence of scrapie and maedi. These restrictions are still prevailing and, therefore, moving rams between farms is currently prohibited in many regions, reducing the effectiveness of the ram circles. As a consequence, the use of AI increased to about 3.5 per cent during the last decade [6-11], emphasizing the need to use more automatic systems for semen processing at the AI-stations. Therefore, in 2001 a field trial based on do-it-yourself (DIY) inseminations [12] was conducted with the aim to compare fertility results after cervical AI with minitubes, thawed at 70°C for 8 sec, and conventional mini straws (0.25 ml, IMV, L'Aigle, France), thawed either at 70°C for 5 sec, 50°C for 9 sec or 35°C for 12 sec. The results showed that cervical AI using minitubes resulted in the highest overall lambing rates and was superior to mini straws (approximately 5 to 10% units) independent of thawing procedure used. The lowest lambing rates, however, were achieved using mini straws thawed at 70°C for 5 sec. Many of the farmers participating in that field trial complained that the combination of short thawing time and high temperature made it difficult to handle the thawing of the thin mini straws in a proper way. The use of mini straws, however, would allow automatic filling and sealing of ram semen at AI-stations as well as more efficient storage in liquid nitrogen containers. Therefore, the superior fertility results were evaluated carefully in relation to the possible application of a more rational semen production and simplified semen handling at AI when using mini straws thawed at 35°C. Consequently, from 2002 freezing of ram semen in mini straws and subsequent thawing at 35°C was considered as the method of choice in Norway.

Successful application of AI in sheep also depends upon the availability of a cheap and effective insemination technique. To attain this goal, an AI technique had to be established that easily could be applied under field conditions by the farmers themselves. Vaginal insemination [13] is easy to perform because only an insemination pipette is required. A Norwegian field trial based on DIY inseminations and performed in 2002 [14] compared vaginal and cervical deposition of frozen-thawed semen, and found somewhat higher fertility results (circa 5% units) for cervical deposition compared to vaginal with a significant difference in the overall lambing rate. However, the study also revealed significant effects both of the farmers performing the inseminations as well as of an interaction between farmer and deposition site. Four out of ten farmers had numerically better results after vaginal compared to cervical deposition, while five farmers had the opposite results. Based on these results, vaginal insemination, being a simple, less costly and less time consuming technique, was recommended for routine use for AI of ewes with frozen-thawed semen in Norway starting in 2003.

Despite the above described, recommended change of semen package and deposition site applied in Norway, the influence on fertility of different straw types and thawing temperatures using vaginal insemination remains to be established.

Therefore, the aim of the present study was to compare, under field conditions, the fertility results after vaginal insemination of ewes using semen frozen in mini tubes and thawed at 70°C for 8 sec with semen frozen in mini straws and thawed at 50°C for 9 sec or at 35°C for 12 sec.

## Methods

### Rams and semen processing

Semen from 7 mature Norwegian Crossbred rams with known fertility was used for AI. The animals were owned by the Norwegian Association of Sheep and Goat Breeders (NSG) and housed at the NSG Semin AI station in south-eastern Norway, near Hamar, a region with inland climate.

Semen was collected three to four times weekly per ram during the breeding season (November–December, Northern Hemisphere) using an artificial vagina. Each ejaculate was collected in a pre-warmed graduated glass vial. Semen quality was assessed, and to be accepted as a donor, every ram had to fulfil the following demands concerning semen quality: volume ≥ 0.5 ml, macroscopic good visual mass activity, sperm concentration ≥ 3 × 10^9^/ml, progressive sperm motility ≥ 75%, and normal sperm morphology ≥ 90%. Motility was assessed subjectively and membrane integrity as well as morphology evaluated after eosin-nigrosin staining. During the whole production period volume, mass activity, sperm concentration and motility were evaluated routinely, while sperm morphology was only assessed occasionally. Sperm concentration was estimated with a spectrophotometer (Accucel, IMV, L'Aigle France), which was calibrated for ram semen. Using a phase contrast microscope sperm motility was assessed at 200 × magnification and morphology at 400 × magnification.

The ejaculates were placed in a water bath (35°C) immediately after collection, and semen quality was assessed. Within 10 minutes after collection each ejaculate was diluted 1+4 to 1+6 with a 35°C warm milk-based extender (E 1), prepared from non-fatty milk powder (11% w/v) and distilled water, heated to 95°C for 10 min, and then cooled to room temperature before egg yolk (5%; w/v), penicillin and streptomycin was added. Each glass with the extended semen was wrapped with tissue paper to protect against cold shock. The semen was then cooled to 5°C during approximately 30 minutes by placing it in a room at that temperature. The cooled semen was then diluted 1+1 with an extender (E 2) of equal temperature. Extender 2 was the same as E 1, but with the addition of glycerol (14%; v/v) resulting in a final glycerol concentration of 7%. Addition of E 2 was performed stepwise during a couple of minutes. After this second dilution the semen was kept at 5°C to allow glycerol equilibration and adaptation to this temperature for 90 to 120 minutes. Then the semen was re-concentrated by centrifugation (1000 g/10 minutes) removing enough supernatant to yield a calculated final sperm concentration of about 1000 × 10^6^/ml. From the cooled and re-concentrated semen one-third part was used to manually fill aliquots of 0.2 ml into minitubes (0.25 ml, Minitüb, Tiefenbach, Germany), and the remaining part was used for automatic filling (MRS 3, IMV, L'Aigle, France) of mini straws (0.25 ml, IMV, L'Aigle, France) resulting in an insemination dose of 200 × 10^6 ^spermatozoa. The minitubes were sealed with plastic balls, while the open end of the mini straws were welded automatically using ultra sound. Freezing was performed in a programmable freezer (Digicool 5300, IMV, L'Aigle France) where the temperature decreased to -10°C at a rate of 5°C/min and from -10°C to -130°C at 60°C/min. Thereafter the straws were transferred to liquid nitrogen. From each batch and each ram one straw was thawed, and only batches showing at least 50% progressive motility were approved for use.

### Ewes and artificial insemination

During a period of about five weeks, from the middle of November to the middle of December 2004, a total of 719 Norwegian Crossbred ewes were inseminated artificially. The ewes were between six months and six-and-a-half years old at the time of insemination and housed at 8 different farms that were located in the mid part of Norway, in a region (Trøndelag) with mostly coastal climate. All farms received semen doses from all rams. The number of semen doses produced from each ram, however, was not identical. In average 103.7 AI-doses, with a range from 31 to 179, were used from each ram. In general, the farmers checked oestrus twice daily with a time interval of about 12 hours using a teaser ram fitted with an apron. The ewes were inseminated once and AI was recommended between 12 – 24 hours after detection of standing oestrus.

The farmers involved in the study had all performed inseminations themselves in their flocks during several years, and were, thus, considered as experienced inseminators. In the farms selected for this trial all ewes were inseminated. The ewes were inseminated vaginally with a dose of approximately 200 × 10^6 ^frozen-thawed spermatozoa. The vaginal inseminations were made with an insemination pipette with a diameter of 5 mm and a rounded tip (Minitüb GmbH, Tiefenbach, Germany); without using a speculum, the lips of the vulva were parted and the semen was placed as deeply as possible into the vagina, as described by Fairnie and Wales (1982).

### Thawing procedures

Minitubes were thawed in water in thermos flasks at 70°C for 8 seconds (T70). Mini straws were thawed either at 50°C for 9 sec (S50) or at 35°C for 12 sec (S35). The thawing time was measured with a timer.

### Experimental design

The females were allocated to three parallel groups within each flock based on the two straw types and different thawing temperatures (see thawing procedures and abbreviations above). The ewes were consecutively inseminated when showing natural oestrus as follows: T70, S50, and S35, respectively, and then starting again from the beginning with a new round. For each round semen from a new ram was used.

### Fertility assessments

To detect ewes returning to oestrus, all ewes were checked from day 12 to day 25 after insemination using a teaser ram. Ewes not returning to oestrus were considered pregnant, and recorded as the percentage of ewes that did not return to oestrus at 25 days after AI (25-days NR). Lambing rates (percentage of ewes lambing) were recorded for each farm during the following spring. Moreover, the farmers recorded ewes returning to oestrus later than 25 days after insemination as well as abortions. All recordings were made by the farmers and a written report was forwarded from each farmer.

### Statistical analysis

The binomial response NR rate (not returned to oestrus) and lambing rate were analyzed using a linear logistic model: the genmod procedure for categorical data (Statistical Analysis Systems statistical package, version 6.12; SAS Institute Inc., Cary, NC, USA).

The model chosen was:

log odds of NR and lambing rate

= age of the ewe + farmer + ram + straw type/thawing temperature

where NR and lambing rate for a ewe is either yes or no,

age of ewe at parturition time is: 1, 2, 3, 4 or >4 years,

farmer is one of 8 farmers,

ram is one of 7 different rams used, and

straw type/thawing temperature is either T70, S50 or S35.

The parameters were fitted by the maximum likelihood principle, and effects tested by Chi-Square [15,16]. Significances of main effects (not accounting for other effects) were confirmed by Fisher's exact tests.

Several alternative statistical models were tried, including combinations of main effects with interactions. The results of the chosen model were in accordance with other simpler models, and the ones with interactions that were possible to run. Because of the fact that the farmers both were responsible for the effects of the flock management, for example oestrus check, and also performed the inseminations, the effect of the inseminator could not be included in addition to farmer. To avoid overlapping only the effect of farmer was included in the statistical model. The level of significance was set to 0.05. The contrast between the 3 comparable pairs of thawing procedures (T70-S50; T70-S35; S50-S35) was tested with chi-square both for non-return and lambing rate.

## Results

### P-values

The levels of significance for the effects of age of the ewe, farmer, ram and straw type/thawing temperature are presented in Table 1. Farmer and ram had a significant influence both on the NR- and lambing rates, while age of the ewes had only a significant influence on the NR-rates. No significant main effects were seen for straw type/thawing temperature on fertility.

**Table 1:** P values for the effects of age of the ewe, farmer, ram and straw type/thawing temperature. P values and chi-square (25-days NR- and lambing rates) for the effects of age of the ewe, farmer, ram and straw type/thawing temperature in 719 ewes, aged between six months and six-and-a-half years, inseminated vaginally with 200 × 10^6 ^frozen-thawed spermatozoa using different packages and thawing temperatures, collected from 7 mature rams.

		Chi-square		P	
		
Source of variation	Degrees of freedom	NR	Lambing	NR	Lambing
Age of the ewes	2	10.7	13.2	0.007	0.220
Farmer	7	15.8	18.9	>0.0001	>0.0001
Ram	6	12.7	11.8	0.009	0.002
Straw type/thawing temperature	2	3.8	6.7	0.515	0.450

NR = 25-days non-return

### Fertility

The 25-days NR- and the lambing rates for the different straw types and thawing temperatures are shown in Table 2. The average 25-days NR rate was 61.8%, ranging between 40.0 to 82.4% in the different farms, and the average lambing rate was 57.0% (range 37.0 to 82.4%). The difference between the overall 25-days NR result and the lambing rate was 4.8% units (n = 35). Out of the 35 ewes 31 returned to oestrus later than 25 days. Four ewes (0.6%) aborted and all abortions were found in one farm. The difference between the overall 25-day NR result and the lambing rate varied among farms (0.0–11.1%) and age of the ewes, and was higher among primipari ewes (18.3%) compared to pluripari ewes (2.3%).

**Table 2:** Fertility results for the different packages and thawing temperatures. Non-return (25-days NR) and lambing rates for different packages and thawing temperatures after vaginal insemination of 719 ewes, aged between six months and six-and-a-half years, with 200 × 10^6 ^frozen-thawed spermatozoa collected from 7 mature rams.

		Non return		Lambing	
		
Straw type/Thawing temperature	Number of inseminations	Number	Percentage	Number	Percentage
T 70	234	148	63.2	133	56.8
S 50	240	143	59.6	132	55.0
S 35	245	153	62.5	145	59.2
Total	719	444	61.8	410	57.0

T70 = Minitubes thawed at 70°C for 8 sec

S50 = Mini straws thawed at 50°C for 9 sec

S35 = Mini straws thawed at 35°C for 12 sec

### Ewe, ram and farmer

On average 90 ewes, with a range from 40 to 160, were inseminated per farm. The average time from detection of standing heat to insemination was approximately 18 hours with a range from 10 to 25 hours. The average number of lambs per ewe was 1.5 for primipari ewes (n = 65), 1.8 for pluripari ewes (n = 345), and 1.75 over all in the study (n = 410).

The fertility results for the different rams varied between 50.0 and 74.3% (average 61.8) for the 25-days NR rate and between 42.8 and 68.2% (average 57.0) for the lambing rate. The difference between the 25-days NR and the lambing rate varied between 0.0 and 7.3% units for the individual rams.

### Pair wise comparison

P values and chi-square for the fertility results, when comparing the three different straw types/thawing temperatures pair wise are shown in Table 3. No significant differences could be seen between the fertility rates

**Table 3:** Comparison of fertility results using different packages and thawing temperatures. P values and chi-squared (25-days NR- and lambing rates) for the comparison of fertility results using different packages and thawing temperatures after vaginal insemination of 719 ewes, aged between six months and six-and-a-half years, with 200 × 10^6 ^frozen-thawed spermatozoa collected from 7 mature rams.

		Chi-Square		P	
		
Comparison between minitubes and mini straws	Degrees of freedom	NR	Lambing	NR	Lambing
T 70 – S 50	1	1.2	0.3	0.3	0.6
T 70 – S 35	1	0.1	0.5	0.8	0.5
S 50 – S 35	1	0.7	1.6	0.4	0.2

T70 = Minitubes thawed at 70°C for 8 sec

S50 = Mini straws thawed at 50°C for 9 sec

S35 = Mini straws thawed at 35°C for 12 sec

NR = 25-days non-return

## Discussion

No significant effect on fertility, expressed as 25-days non-return- or lambing rate, was seen in the present study for straw type/thawing temperature. A significant influence of farmer, ram, and age of the ewes was seen on the fertility results. Semen filled in mini straws and thawed at 35°C gave numerically the highest lambing rate with 59.2%, while the 25-days NR rate was 62.5%. The overall national fertility results in the same season (2004), only available as 25 days NR rates, were 61.3% (n = 14327) and in accordance with the results obtained in this study [11,17].

In the literature, the influence of different packaging on sperm survival has been studied, using identical thawing procedures by Maxwell et al. [18], while Söderquist et al. [19] compared thawing of ram semen frozen in mini straws at 70, 50 and 35°C and found that the post-thaw sperm motility, as well as the percentage of spermatozoa depicting intact membranes, were significantly higher in straws thawed at 70°C compared to at 35°C. No statistically significant difference was, however, found for these parameters when the results from thawing at 70°C and 50°C were compared. Later, in a field trial with a limited number of inseminations, Söderquist et al. [20] reported that thawing of mini straws at 50°C for 9 sec, instead of 70°C for 5 sec, did not seem to further affect neither fertility nor fecundity.

In a recent study Paulenz et al. [12] thawed mini tubes at 70°C for 8 sec (T70) and mini straws either at 70°C for 5 sec (S70), 50°C for 9 sec (S50) or 35°C for 12 sec (S35). No significant effects were seen for straw type/thawing temperature after cervical insemination with 200 × 10^6 ^spermatozoa on fertility expressed as 25-days non-return- or lambing rate. There was, however, a significantly higher lambing rate for T70 compared to S35 but not compared to S50. The highest lambing rate was achieved for mini tubes (T70). However, in the present study the highest results were seen using mini straws (S35), but no significant difference was found when comparing T70 and S35. The discrepancy between these two studies is unclear, but the different deposition sites may have contributed to the result.

Minitubes are similar to mini straws with the exception of their surface-to-volume ratios, the thickness of the material in the tube and the way the ends are sealed after filling the straws. Especially the larger surface-to-volume ratio of the mini straws may have implications for both the freezing and the thawing rates. Therefore, it would be optimal to include also the freezing rate, when comparing different straw types. In a field trial under practical conditions, however, only a limited number of variables could be tested, why we in the present study chose to use the routine freezing rate for both straw types. Further studies are needed to assess the influence of different freezing rates on different packages.

The variation in fertility results between the individual farmers in the NR rates as well as in lambing rates are in accordance with results from our earlier studies [12,14,20-22]. These differences among the farmers represent not only the level of technical skill of insemination itself but also flock conditions like detection of heat and oestrus symptoms [23]. Other factors, like e.g. feeding or animal handling, might also have contributed to the differences seen herein. On the whole, the above mentioned factors are of great importance for successful AI results underlining the need for a continuous guidance and further training of the DIY-farmers in order to optimise the conditions on the farms.

There were significant differences in the NR and lambing rates of ewes inseminated with semen from different rams. Such variations in fertility of rams are well documented and have been reported using fresh semen by Salamon and Robinson [24] following cervical insemination and Paulenz et al. [21] following vaginal insemination. For the use of frozen-thawed semen sire effects are described by Maxwell [25], Eppleston et al. [26,27], and Eppleston and Maxwell [28] following laparoscopic insemination, by Colas [29], Windsor [30], and Söderquist et al. [20] following cervical insemination, and by Paulenz et al. [14] following vaginal insemination. The rams used as donors in this study were selected after an estimation of a breeding value that was based on the results of natural mating in the previous season. Though the breeding value includes fertility as an important factor, the variation observed between the seven rams emphasises the need for improved methods for the selection of AI-rams.

It is noteworthy that the difference between the overall 25-day NR result and the lambing rate was about twice as high in the present study as found in our earlier studies [12,14,22]. This increased value was obviously caused by a much higher difference seen among primipari ewes (18.3%) compared to pluripari ewes (2.3%). The reason for this is not fully understood, but several factors may be of importance, e.g. insufficient oestrous control in some farms, incapacity to show oestrus caused by low ranking, hidden anatomical or physiological disorders and defects.

It is desirable to find a more rational procedure for semen production at the AI station, but also to introduce a simple and safe thawing procedure that could be applied under practical field conditions in Nordic countries, similarly on a large scale by all farmers. The use of mini straws allows automatic filling and sealing of ram semen at AI-stations as well as more efficient storage in liquid nitrogen containers. Furthermore, the use of mini straws and thawing them at 35°C diminishes the risk for maltreatment at AI such as overheating the semen [19]. In addition, vaginal deposition of frozen-thawed semen simplifies the AI technique, being simple, less costly and less time consuming compared to others, also bringing into focus the animal welfare aspects at AI.

## Conclusions

The use of vaginal deposition of frozen ram semen containing approximately 200 × 10^6 ^spermatozoa, filled in mini straws and thawed at 35°C is a simplified technique that under field conditions and used on a DIY basis gives acceptable lambing rates in Norway. Furthermore, freezing of ram semen in mini straws would also result in a more rational semen production and simplified semen handling at the AI centres.

## Competing interests

The author(s) declare that they have no competing interests.

## Authors' contributions

HP was main responsible and involved in all parts of the study. LS participated in all parts of the study but not in the statistical analysis. TA participated in the design of the study and performed the statistical analysis. All authors read and approved the final manuscript.
